# Sustainable Recovery of Critical Minerals from Wastes by Green Biosurfactants: A Review

**DOI:** 10.3390/molecules30112461

**Published:** 2025-06-04

**Authors:** Bita Deravian, Catherine N. Mulligan

**Affiliations:** Department of Building, Civil and Environmental Engineering, Concordia University, 1455 de Maisonneuve Blvd. W., Montreal, QC H3G 1M8, Canada; deravianbita@gmail.com

**Keywords:** biosurfactants, critical minerals, metal recovery, resource circularity, green analytical chemistry, heavy metals, sustainable extraction

## Abstract

Biosurfactants have emerged as promising agents for environmental remediation due to their ability to complex, chelate, and remove heavy metals from contaminated environments. This review evaluates their potential for recovering critical minerals from waste materials to support renewable energy production, emphasizing the role of biosurfactant–metal interactions in advancing green recovery technologies and enhancing resource circularity. Among biosurfactants, rhamnolipids demonstrate a high affinity for metals such as lead, cadmium, and copper due to their strong stability constants and functional groups like carboxylates, with recovery efficiencies exceeding 75% under optimized conditions. Analytical techniques, including Inductively Coupled Plasma Mass Spectrometry (ICP-MS), Fourier-Transform Infrared spectroscopy (FTIR), and Scanning Electron Microscopy (SEM), are instrumental in assessing recovery efficiency and interaction mechanisms. The review introduces a Green Chemistry Metrics Framework for evaluating biosurfactant-based recovery processes, revealing 70–85% lower Environmental Factors compared to conventional methods. Significant research gaps exist in applying biosurfactants for extraction of metals like lithium and cobalt from batteries and other waste materials. Advancing biosurfactant-based technologies hold promise for efficient, sustainable metal recovery and resource circularity, addressing both resource scarcity and environmental protection challenges simultaneously.

## 1. Introduction

### 1.1. Critical Minerals in the Context of Global Sustainability

The global community faces unprecedented challenges in securing adequate supplies of critical minerals to support the clean energy transition [1,2]. The rapid expansion of renewable energy technologies, electronics, and electric vehicles has dramatically increased the demand for these essential materials [3,4]. Critical minerals, including rare earth elements (REEs), lithium, cobalt, nickel, and others, form the backbone of modern technologies that underpin the energy transition, including electric vehicles, wind turbines, solar panels, and energy storage systems [5,6]. The International Energy Agency projects that the demand for critical minerals for clean energy technologies could increase by 400–600% by 2040, depending on the pace of the energy transition [3]. Specifically, demand for lithium and cobalt could grow by over 2000% and 600%, respectively, in scenarios aligned with the Paris Agreement targets [4]. Such growth places significant strain on conventional mining systems, which are often geographically concentrated, environmentally intensive, and socially contentious. In this context, developing alternative, more sustainable recovery pathways—particularly from secondary sources such as industrial wastes, spent batteries, and electronic scrap—has become a priority for governments, researchers, and industry alike.

These challenges call for not only innovation in recovery technologies but also a paradigm shift in how resource management is approached. Integrating principles of green chemistry and circular economy into mineral recovery processes can help reduce the environmental footprint of extraction while enhancing supply chain resilience.

### 1.2. Waste as a Secondary Resource for Critical Minerals

The accelerating global demand for critical minerals is driving the urgent need to identify alternative and more sustainable resource streams. Industrial residues, spent batteries, electronic waste (e-waste), and mine tailings are increasingly recognized as valuable secondary sources for critical mineral recovery, offering a pathway to reduce dependence on primary extraction. These waste streams often contain metal concentrations comparable to or exceeding those of natural ores, highlighting their substantial untapped potential for enabling circular resource flows [7].

Notably, e-waste has emerged as one of the fastest-growing waste categories worldwide, reaching an estimated 62 million metric tons in 2022, with projections surpassing 82 million metric tons by 2030 [8]. Simultaneously, the end-of-life volume of lithium-ion batteries is expected to rise sharply, exceeding 11 million metric tons globally by 2030 [8].

Mining residues, including tailings and waste rock, also represent latent resources, containing significant concentrations of valuable metals that were previously deemed uneconomical to recover. Advances in extraction technologies and shifting market dynamics have renewed interest in harnessing these materials for critical mineral recovery initiatives [9].

Conventional approaches to resource recovery from these waste streams—such as pyrometallurgical smelting, hydrometallurgical leaching with inorganic acids, and emerging solvometallurgical and bioleaching methods—have demonstrated technical viability at scale [10]. However, these processes frequently entail high energy demands, reliance on hazardous chemicals, and complex separation challenges, raising concerns over their environmental footprint and economic sustainability [11].

Against this background, the development of greener and more selective recovery technologies has gained prominence. Among these, biosurfactant-based extraction methods have emerged as a promising alternative, leveraging biologically produced surface-active compounds characterized by selective metal binding, low toxicity, and inherent biodegradability [12]. Integrating biosurfactants into critical mineral recovery frameworks offers an opportunity to advance more sustainable, circular, and environmentally benign resource utilization.

### 1.3. Green Analytical Chemistry in Metal Recovery

Green analytical chemistry (GAC) represents a paradigm shift in how analytical and recovery processes are designed and implemented [13]. The principles of GAC aim to reduce environmental impact while maintaining or improving analytical performance [14]. In the context of critical mineral recovery, GAC principles guide the development of more sustainable extraction, separation, and analytical methodologies [15].

The 12 principles of Green Chemistry, when applied to analytical processes and metal recovery, emphasize waste prevention, less hazardous chemical synthesis, safer chemicals and processes, renewable feedstocks, catalysis, real-time analysis for pollution prevention, and inherently safer chemistry [15,16]. These principles have driven interest in biosurfactants as green alternatives to conventional extraction agents such as synthetic surfactants, aligning with the transition toward more sustainable analytical and remediation technologies [16].

GAC has revolutionized sample preparation methodologies by integrating sustainability, efficiency, and safety into procedures while meeting analytical and economic requirements [17]. Properly incorporating green chemistry into sample preparation techniques and remediation can significantly reduce or eliminate the use of hazardous substances, such as toxic solvents, thereby minimizing risks to human health and contributing to environmental protection [17,18].

### 1.4. Biosurfactants as Green Alternatives for Metal Recovery

Biosurfactants represent an emerging class of green chemicals with significant potential for metal recovery applications [19]. These amphiphilic compounds, produced primarily by microorganisms, offer several advantages over synthetic surfactants, including lower toxicity, higher biodegradability, better environmental compatibility, and production from renewable resources [20,21].

Initially studied for their applications in environmental remediation of metal-contaminated sites, biosurfactants have demonstrated remarkable capabilities for mobilizing and removing heavy metals from soils and sediments [22,23]. Their success in environmental remediation contexts has prompted investigations into their potential for resource recovery applications, particularly for critical minerals from waste materials [24].

Several classes of biosurfactants show promise for metal recovery, including glycolipids (rhamnolipids and sophorolipids), lipopeptides (surfactin), and polymeric biosurfactants (emulsan) [25]. Among these, rhamnolipids produced by *Pseudomonas aeruginosa* have shown efficacy for metal recovery due to their strong metal-binding capabilities and favorable physicochemical properties [26]. The application of biosurfactants for metal recovery represents a convergence of environmental remediation and resource recovery technologies, potentially offering sustainable pathways to address both metal contamination and resource scarcity challenges simultaneously [27].

### 1.5. Need for Standardized Green Chemistry Metrics

Despite the growing body of research on biosurfactant-mediated metal recovery, there remains a lack of standardized metrics to evaluate the “greenness” and sustainability of these processes. Current studies often focus on recovery efficiency without comprehensive assessment of environmental impact, resource consumption, energy requirements, or life cycle considerations [28,29].

This gap hinders objective comparison between different biosurfactant-based approaches, meaningful comparison with conventional recovery methods, systematic optimization toward greener processes, regulatory acceptance and industrial adoption, and alignment with circular economy principles [28]. Without standardized metrics, the true environmental benefits of biosurfactant-based approaches remain difficult to quantify and communicate to stakeholders [29]. A comprehensive Green Chemistry Metrics Framework specifically tailored to biosurfactant-based metal recovery would address these limitations, providing a systematic approach to evaluate and advance these promising technologies within the broader context of sustainable resource management.

## 2. Critical Minerals for Renewable Energy Technologies

### 2.1. Definition and Classification of Critical Minerals

Critical minerals are defined as raw materials of high economic importance that face significant supply risks [30]. Various governmental and international organizations maintain lists of critical minerals based on different criteria, including economic value, supply risk, and strategic importance. The European Commission’s list includes 30 materials, while the U.S. Department of the Interior identifies 50 minerals as critical [31,32].

For renewable energy applications, several mineral groups are particularly significant. Battery minerals include lithium, cobalt, nickel, manganese, and graphite, which are essential components of lithium-ion batteries used in electric vehicles and energy storage systems [7]. The criticality of these minerals stems from their essential functions in clean energy technologies, limited substitutability, and concentrated supply chains. For instance, cobalt serves as a cathode material in lithium-ion batteries, enhancing stability and energy density, with few viable alternatives that maintain equivalent performance [33].

### 2.2. Global Demand and Supply Challenges

The clean energy transition is driving unprecedented demand for critical minerals. Under the Sustainable Development Scenario, the International Energy Agency projects that mineral demand for clean energy technologies could increase by 400–600% by 2040 [34]. Electric vehicles and battery storage account for about half of this growth, with their mineral intensity being significantly higher than conventional technologies [35].

Supply challenges include geological scarcity, declining ore grades, geopolitical concentration, long lead times for new mining projects, environmental and social impacts of extraction, market volatility, and processing bottlenecks [36]. For example, developing new mining operations typically requires 10 to 15 years from discovery to production, creating significant lags in supply responses to increased demand [37].

The geographical concentration of both reserves and processing capabilities presents additional vulnerabilities. China processes approximately 35% of global nickel, 59% of lithium, 68% of cobalt, and 85% of rare earth elements, creating potential bottlenecks and strategic considerations in supply chains. These figures underscore the importance of developing diversified supply chains and alternative recovery technologies to reduce dependence on geographically concentrated sources [38].

### 2.3. Critical Minerals in Waste Streams

Various waste streams contain significant concentrations of critical minerals that can potentially be recovered. Electronic waste represents one of the fastest-growing waste streams globally, with approximately 53.6 million metric tons generated in 2020 and projected to increase to 74.7 million metric tons by 2030. Circuit boards in e-waste can contain 40–50 times the concentration of gold and 10–20 times the concentration of copper compared to primary ores [39,40].

The global volume of spent lithium-ion batteries is projected to reach 2 million metric tons annually by 2030, representing a significant repository of critical minerals [41]. Depending on the battery chemistry, these batteries contain cobalt (5–20%), lithium (2–7%), nickel (5–10%), and manganese (5–15%) [42]. The diversity of battery chemistries presents both challenges and opportunities for recovery processes, requiring flexible approaches capable of handling variable compositions [43].

Mining wastes, including tailings and waste rock, often contain residual concentrations of target metals as well as other valuable elements not initially targeted for recovery. Historical tailings may contain significant amounts of critical minerals that were not economically valuable at the time of initial processing but are now in high demand [44]. The concentration, physical state, and chemical associations of critical minerals in these waste streams significantly influence the selection of appropriate recovery technologies, including biosurfactant-based approaches [45].

## 3. Green Biosurfactants: Properties and Production

### 3.1. Biosurfactant Classification and Structure

Biosurfactants are surface-active compounds produced by microorganisms, including bacteria, fungi, and yeasts [46]. Like synthetic surfactants, biosurfactants contain both hydrophilic and hydrophobic moieties, allowing them to reduce surface tension, increase solubility of hydrophobic substances, and form micelles [47].

Biosurfactants are classified based on their chemical composition and microbial origin into several major categories. Glycolipids include rhamnolipids (produced by *Pseudomonas aeruginosa*), sophorolipids (produced by *Candida* species), and trehalose lipids (produced by *Rhodococcus* species) [48]. Lipopeptides and lipoproteins include surfactin, iturin, and fengycin (produced by *Bacillus* species) [49]. Other categories include fatty acids, phospholipids, and neutral lipids produced by various microorganisms, polymeric biosurfactants such as emulsan and alasan (produced by *Acinetobacter* species), and particulate biosurfactants including vesicles and whole microbial cells [50].

Among these classes, glycolipids—particularly rhamnolipids—have shown the most promise for metal recovery applications due to their strong metal-binding capabilities and favorable physicochemical properties [51]. Table 1 presents the key properties of the most relevant biosurfactants for critical mineral recovery, highlighting their structural, metal-binding, environmental, and production characteristics.

### 3.2. Production Methods and Microbial Sources

Biosurfactants are produced through microbial fermentation processes using various carbon sources. The primary bacterial sources include *Pseudomonas aeruginosa* for rhamnolipids and *Bacillus subtilis* for surfactin and other lipopeptides [52]. Other bacterial producers include the *Acinetobacter*, *Rhodococcus*, and *Arthrobacter* species [53]. Fungal and yeast sources include *Candida bombicola* (*Starmerella bombicola*) for sophorolipids, and various *Candida* and *Yarrowia* species for mannosylerythritol lipids and other glycolipids [54].

Despite their advantages, the commercial production of biosurfactants faces challenges, including low yields, high purification costs, and substrate specificity of producing organisms [55]. Recent advances in genetic engineering, bioreactor design, and downstream processing have begun to address these limitations, improving production efficiency and reducing costs [56].

### 3.3. Environmental Advantages of Biosurfactants

Compared to synthetic surfactants, biosurfactants offer several environmental advantages that align with green chemistry principles. Their high biodegradability reduces environmental persistence and accumulation, with most biosurfactants degrading by more than 80% within 28 days under aerobic conditions [57].

Life cycle assessments indicate that biosurfactants generally have lower carbon footprints than synthetic surfactants, particularly when produced from waste substrates [58]. These advantages make biosurfactants particularly suitable for environmental applications, including metal recovery from waste streams, where minimizing additional environmental impacts is a priority [59].

### 3.4. Physicochemical Properties Relevant to Metal Recovery

Several physicochemical properties of biosurfactants are particularly relevant to their metal recovery capabilities. The critical micelle concentration (CMC) refers to the concentration at which biosurfactants begin to form micelles, typically ranging from 5 to 200 mg/L for rhamnolipids, compared to 1300–1500 mg/L for synthetic surfactants like sodium dodecyl sulfate (SDS) [60]. Lower CMC values indicate greater efficiency at lower concentrations, reducing chemical inputs in recovery processes [61].

Biosurfactants can reduce the surface tension of water from 72 mN/m to 25–30 mN/m, facilitating interactions with hydrophobic surfaces and enhancing metal mobilization [62]. The presence of carboxyl, hydroxyl, and phosphate groups in biosurfactants enables various metal-binding mechanisms, including complexation, chelation, and ion exchange [63]. Many biosurfactants maintain stability across a wide pH range (4–10), though their metal-binding capabilities are often pH-dependent due to the protonation/deprotonation of functional groups [64]. Different biosurfactants exhibit varying affinities for specific metals, with stability constants (log K) ranging from four to nine for various metal–rhamnolipid complexes, offering potential for selective recovery [65]. These properties collectively determine the effectiveness of biosurfactants for recovering specific metals from complex waste matrices and influence the design of biosurfactant-based recovery processes [64,65]. Table 1 presents a comprehensive overview of these properties for the most relevant biosurfactants in metal recovery applications; this table illustrates the key properties of biosurfactants that make them suitable for critical mineral recovery. Rhamnolipids show particular promise with their low CMC values (5–200 mg/L) enabling effectiveness at lower concentrations than conventional surfactants. The ability of these biosurfactants to reduce surface tension to 25–35 mN/m enhances interaction with metal-containing matrices, while their high biodegradability (>80% within 28 days) offers environmental advantages. The optimal pH ranges directly affect metal binding capacity, with rhamnolipids demonstrating excellent stability across varying conditions. Although rhamnolipids appear most promising for metal recovery applications, surfactin and sophorolipids also exhibit valuable characteristics that may be advantageous for specific recovery scenarios from different waste streams.

## 4. Mechanisms of Biosurfactant–Metal Interactions

### 4.1. Complexation and Chelation

Complexation and chelation represent primary molecular mechanisms by which biosurfactants interact with metal ions. These interactions are driven by the presence of electron-donating functional groups, enabling biosurfactants to form stable complexes with various metals [66,67]. Understanding these molecular-level processes is critical for optimizing the use of biosurfactants in critical mineral recovery from waste streams.

#### 4.1.1. Chemical Structure and Metal-Binding Functional Groups

Biosurfactants such as rhamnolipids, surfactin, and sophorolipids possess amphiphilic structures, with distinct hydrophilic and hydrophobic domains that facilitate metal binding [66,68]. Rhamnolipids consist of one or two rhamnose sugar moieties linked to β-hydroxy fatty acid chains, where functional groups such as carboxyl (-COOH) groups on fatty acids and hydroxyl (-OH) groups on sugar moieties are crucial for coordinating metal ions [66].

The carboxyl groups act as primary binding sites by donating electron pairs to metal cations, while the hydroxyl groups can further stabilize the interaction through hydrogen bonding or secondary coordination. These functional groups, spatially arranged within the amphiphilic structure, allow biosurfactants to simultaneously interact with multiple metal ions, promoting stable complex formation [66,67].

**Figure 1:**
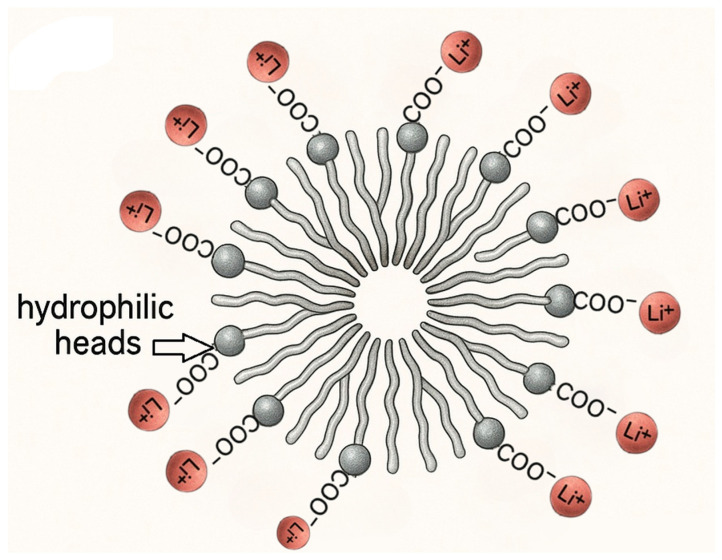
Schematic of biosurfactant functional groups and micelle formation with metal binding sites (carboxyl and hydroxyl groups) [69].

A schematic illustration of these functional groups and their role in biosurfactant–metal complexation is presented in Figure 1, highlighting the interaction between molecular structure and micelle entrapment mechanisms.

#### 4.1.2. Mechanism of Metal Complexation

Metal complexation by biosurfactants typically initiates through the deprotonation of carboxyl groups under neutral to slightly alkaline conditions, resulting in negatively charged carboxylate (-COO^−^) groups that can attract and bind metal cations [70]. Electrostatic attraction initially drives the interaction, which is then stabilized through coordinating covalent bonding involving oxygen donor atoms [71].

Studies have shown that rhamnolipid–metal binding is significantly affected by pH, with optimal heavy metal removal typically occurring under alkaline conditions (pH 8–11) where carboxyl groups are fully deprotonated [70]. The pH not only influences the ionization state of the biosurfactant but also affects the speciation of the metal ions, thereby determining the overall complexation efficiency [66,67].

#### 4.1.3. Spectroscopic Evidence for Metal–Biosurfactant Complexation

The formation of metal–biosurfactant complexes has been confirmed through various spectroscopic methods. FTIR reveals significant shifts in the asymmetric (νas COO^−^) and symmetric (νs COO^−^) stretching vibrations of carboxylate groups upon metal binding [68].

Advanced X-ray absorption spectroscopic techniques such as X-ray Absorption Near Edge Structure (XANES) and Extended X-ray Absorption Fine Structure (EXAFS) provide atomic-level insights into coordination environments of metals complexed with various ligands [72]. These techniques can reveal changes in metal oxidation states and coordination geometry upon complexation with biosurfactants, confirming the binding modes and metal-oxygen bond distances in rhamnolipid-metal complexes.

The understanding of these molecular-level interactions provides a foundation for developing targeted biosurfactant formulations for critical mineral recovery from various waste streams. Laboratory studies have demonstrated the selective binding capabilities of rhamnolipids toward valuable metals, suggesting promising applications in resource recovery and environmental remediation technologies [70,71].

### 4.2. Micelle-Mediated Solubilization

Above their critical micelle concentration (CMC), biosurfactants such as rhamnolipids and sophorolipids self-assemble into micelles, which play a significant role in enhancing the solubilization of critical metals from waste materials, including spent batteries, electronic waste (WEEE), and mining residues. These micelles, characterized by hydrophilic exteriors and hydrophobic cores, enable electrostatic interactions and complexation with metal ions, thereby increasing their solubility and mobility in aqueous systems [73]. A schematic representation of this mechanism is provided in Figure 1.

Micelle-mediated solubilization typically proceeds through four key stages: (1) formation of micelles when biosurfactant concentration exceeds the CMC; (2) binding of metal ions to functional groups (e.g., carboxylate, hydroxyl) on micelle surfaces; (3) incorporation or encapsulation of the metal–biosurfactant complex within the micellar structure; and (4) stabilization of solubilized metals in the aqueous phase, facilitating subsequent separation and recovery [73].

Recent studies have demonstrated the efficacy of this approach in various contaminated matrices. A biosurfactant derived from *Candida guilliermondii* achieved removal efficiencies of 98.9% Zn, 89.3% Fe, and 89.1% Pb from industrially impacted soils [74]. Similarly, a lipopeptide biosurfactant from *Bacillus* removed 99.93% Cd, 97.7% Pb, 89.5% Mn, and 75.5% Hg from multi-metal aqueous solutions when applied at twice the CMC [75]. Sophorolipid-based washing of arsenic-contaminated soils yielded approximately 91% As removal, while rhamnolipids have been used to extract Cu, Zn, Pb, and As from mine tailings and polluted soils [69].

**Figure 2:**
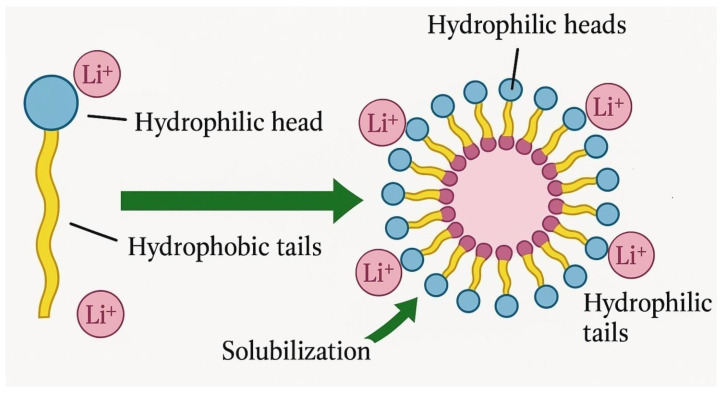
Mechanism of lithium ion (Li^+^) solubilization via micelle formation by biosurfactants [69].

Figure 2 demonstrates the micelle-mediated solubilization mechanism, wherein biosurfactants aggregate to form micelles that incorporate lithium ions (Li^+^) through electrostatic interactions. The figure highlights the amphiphilic nature of biosurfactant molecules and their role in enhancing the solubility and transport of metal ions in aqueous solutions.

### 4.3. Ion Exchange and Electrostatic Interactions

Ion exchange represents another significant mechanism for biosurfactant–metal interactions, particularly at solid–liquid interfaces. Biosurfactants can displace metals bound to solid matrices through competitive ion exchange processes, facilitating metal mobilization and recovery [76].

The ion exchange process typically involves adsorption of biosurfactants onto solid surfaces containing bound metals, displacement of metal ions by biosurfactant functional groups or associated counter-ions, release of metals into solution in forms amenable to recovery, and potential formation of subsequent metal–biosurfactant complexes [77].

Electrostatic interactions also play a crucial role, particularly for anionic biosurfactants like rhamnolipids and surfactin. At appropriate pH levels, these biosurfactants carry negative charges that attract positively charged metal ions [78]. Surface potential measurements and zeta potential analyses have confirmed the importance of these electrostatic interactions in metal recovery processes [63].

Sequential extraction is an analytical technique that uses progressively stronger chemical reagents to determine metal distribution across different geochemical phases in soils. Studies have demonstrated that biosurfactants are particularly effective at mobilizing exchangeable, carbonate-bound, and organically bound metal fractions, with lower efficacy for metals bound to oxides or present in residual fractions. For instance, Mulligan et al. showed that rhamnolipids could mobilize 65–85% of the exchangeable and carbonate-bound zinc and copper fractions from contaminated soils, compared to 15–30% of oxide-bound fractions [79].

**Figure 3:**
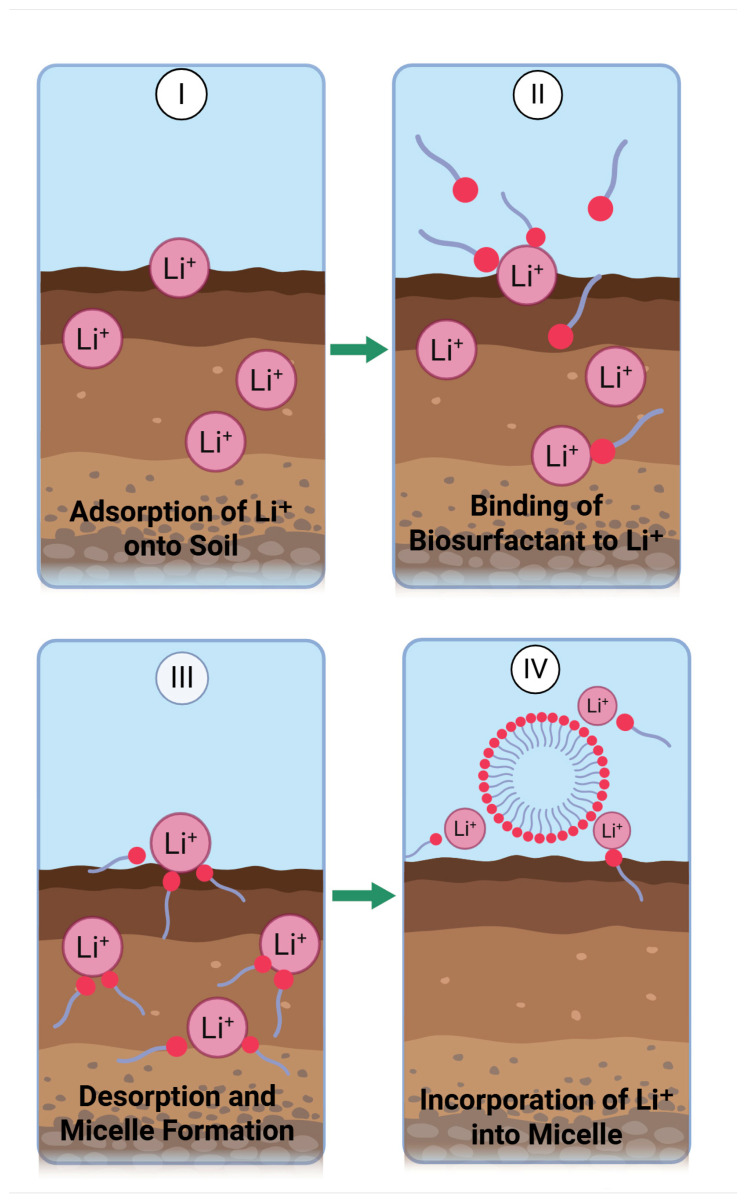
Schematic representation of biosurfactant-mediated lithium ion (Li^+^) recovery from contaminated matrices. (**I**) Adsorption of Li^+^ ions onto soil particles; (**II**) Binding of biosurfactant monomers to Li^+^ ions on the soil surface; (**III**) Desorption and micelle formation with incorporated metal ions; (**IV**) Incorporation of Li^+^ ions into biosurfactant micelles in the aqueous phase. Adapted and modified from [80].

Figure 3 illustrates a generalized, adapted schematic of biosurfactant-mediated lithium recovery from solid matrices, involving sequential processes of surface adsorption, metal–biosurfactant complexation, desorption, and micellar entrapment. Although adapted from [80], this representation integrates updated insights specific to lithium recovery via biosurfactants.

Initially, in step (i), heavy metal ions adsorb onto soil surfaces. Next, in step (ii), monomeric biosurfactants form complexes with these metal ions through sorption and complexation. In stage (iii), the complex of monomers and metals desorb from the solid surface, and then in step (iv), micelles form in solution and the metal/micelle complex can be recovered via ultrafiltration or other processes. This approach offers an environmentally sustainable alternative for remediating metal-contaminated soil or wastes.

## 5. Recovery of Critical Minerals by Biosurfactants

### 5.1. Recovery from Electronic Waste

Electronic waste (e-waste) represents a rich source of critical minerals, including copper, zinc, lead, lithium, cobalt, and nickel. Biosurfactants have shown promise for recovering these metals through various mechanisms [81,82].

Rhamnolipids can recover up to 85% of copper, 75% of lead, and 80% of zinc from printed circuit boards under optimized conditions (pH 5–6, biosurfactant concentration 500–1000 mg/L) [82]. The recovery process typically involves size reduction and physical preprocessing of e-waste, biosurfactant-enhanced leaching under controlled pH conditions, separation of metal-loaded solutions, recovery of metals through precipitation, electrowinning, or adsorption, and recycling of biosurfactants where feasible [81,82].

However, challenges remain in applying biosurfactant-based approaches to complex e-waste matrices, including the presence of flame retardants and other organics that may interfere with recovery, variable composition of e-waste streams, the need for preprocessing to enhance accessibility of target metals, and integration with existing recycling infrastructure.

Table 2 provides critical comparative data on biosurfactant-based and conventional recovery efficiencies for metals from different waste streams. The data demonstrates that while conventional methods generally achieve higher recovery rates (~90–100%) [83,84], biosurfactant approaches—particularly those using rhamnolipids and sophorolipids—offer promising alternatives, achieving recovery efficiencies ranging from 30% to 65% under mild conditions [81,82,85].

These findings emphasize the potential of biosurfactant-based technologies as more sustainable alternatives to conventional extraction methods, while acknowledging the current performance gap that future research must address to enhance their commercial viability in critical mineral recovery applications.

### 5.2. Recovery from Mining Wastes

Mining waste, including tailings, waste rock, and acid mine drainage—are increasingly viewed as valuable secondary resources for critical minerals such as copper, cobalt, nickel, zinc, and rare earth elements (REEs). These wastes, once seen solely as environmental liabilities, now represent a strategic reserve for resource recovery under a circular economy framework [88]. The use of biosurfactants in metal extraction offers promising advantages over conventional acid leaching, including greater selectivity, lower ecotoxicity, and reduced use of hazardous reagents.

Experimental studies have shown that rhamnolipids can recover more than 80% of copper and zinc from sulfidic tailings under optimized conditions—biosurfactant concentrations of 2–5 g/L, pH 4.5–6.0, contact times of 5 days, and liquid-to-solid ratios (L/S) of 10:1 to 20:1 (*v*/*wt*) [89,90].

The advantages of biosurfactant-based approaches for mining waste include a lower environmental impact compared to acid leaching, selective recovery of target metals, potential for in situ application in some contexts, compatibility with existing hydrometallurgical recovery processes, and potential simultaneous environmental remediation benefit [90].

### 5.3. Recovery from Spent Batteries

Spent lithium-ion batteries (LIBs) represent a major secondary source of critical minerals, particularly lithium, cobalt, and nickel. Recovering these metals in an environmentally sustainable way has become increasingly important as conventional acid-based leaching methods raise concerns over waste generation, toxicity, and energy intensity [10].

Biosurfactants offer a low-impact alternative, enabling metal solubilization under milder conditions without the use of corrosive chemicals. Among biosurfactants, rhamnolipids have been most extensively studied for LIB recycling due to their strong complexation affinity, especially for divalent cations like Co^2+^ and Ni^2+^ [71]. Recent leaching studies have shown that rhamnolipid solutions, under optimized conditions (pH 4.0–5.0, 2–5 g/L biosurfactant, 25–30 °C, and 48 h contact time), can achieve recovery rates of up to 90% for cobalt, 85% for nickel, and approximately 75% for lithium from spent lithium-ion batteries [85,87].

Lithium recovery remains more limited compared to cobalt and nickel, largely due to its stronger lattice binding in cathode oxides [87]. However, extraction efficiency can be improved through pH adjustment and extended leaching times. For instance, Alsaqer et al. reported that rhamnolipid leaching at pH~7 achieved 30% Ni and 85% Mo recovery from spent refinery catalysts over a 7-day period [85].

Despite these benefits, several technical challenges remain. These include the need to improve process kinetics, selectively separate metals with similar coordination chemistry (e.g., Co and Ni), and reduce biosurfactant production costs. Ongoing research into biosurfactant recovery and scale-up strategies continue to advance the feasibility of this technology as a viable option for green battery recycling.

## 6. Green Chemistry Metrics Framework for Biosurfactant-Based Metal Recovery

### Conceptual Foundation and Principles

The proposed Green Chemistry Metrics Framework for biosurfactant-based metal recovery is grounded in the principles of Green Analytical Chemistry (GAC) and Life Cycle Assessment (LCA). This framework provides a systematic, quantitative approach to evaluating the environmental impact, efficiency, and sustainability of biosurfactant-based recovery processes for critical minerals [91].

The framework integrates five key domains: environmental impact metrics, resource efficiency metrics, analytical performance metrics, technical feasibility metrics, and economic viability metrics. Each domain comprises specific, measurable indicators that together enable a comprehensive assessment of the “greenness” and long-term sustainability of recovery technologies [92].

This holistic approach addresses a major shortcoming of many current evaluations, namely, a focus solely on recovery efficiency by also considering broader sustainability criteria such as waste generation, energy demand, chemical safety, and biodegradability [93]. By quantifying these trade-offs, the framework facilitates informed decision-making for technology development and process optimization.

The framework aligns closely with the core principles of GAC, particularly those concerning minimal sample preparation, safe reagents, energy efficiency, and operator safety. As shown in Table 3, biosurfactant-based metal recovery systems consistently outperform conventional hydrometallurgical and pyrometallurgical technologies across multiple green metrics. These advantages include lower energy use, fewer hazardous reagents, reduced environmental footprint, and enhanced operator safety while maintaining effective recovery yields.

## 7. Analytical Methods for Characterizing Biosurfactant–Metal Interactions

The effective application of biosurfactants in metal recovery processes relies heavily on a detailed understanding of their interaction mechanisms with metal ions. Recent research has increasingly employed advanced analytical methods to elucidate the physicochemical behaviors governing these interactions. Among these, spectroscopic and microscopic techniques have emerged as the primary tools for probing complexation mechanisms, functional group involvement, surface modifications, and overall recovery performance.

### 7.1. Spectroscopic Methods

FTIR has been particularly effective in identifying the chemical groups involved in biosurfactant–metal binding. In a study by Ravindran et al. [75], a thermostable lipopeptide biosurfactant produced by *Bacillus* species was tested for its ability to bind and remove heavy metals including Pb^2+^, Hg^2+^, Cd^2+^, and Mn^2+^. FTIR spectra of the biosurfactant–metal complexes showed distinct shifts in the absorption bands corresponding to carboxyl and amide groups, confirming their direct involvement in chelation. The biosurfactant achieved removal efficiencies between 85% and 100%, highlighting the strong affinity of its functional groups for multiple metal ions. In addition, ICP-MS analysis provided precise quantification of residual metal concentrations before and after biosurfactant treatment, validating the recovery performance.

### 7.2. Microscopic and Surface Analysis Techniques

To complement these molecular insights, a study also employed transmission electron microscopy (TEM) and energy-dispersive X-ray spectroscopy (EDS) to analyze the structure and elemental composition of the biosurfactant–metal complexes. TEM imaging revealed the formation of spherical aggregates, indicating precipitation of the metal-bound biosurfactant complexes, while EDS confirmed the incorporation of the heavy metals within the aggregates [75]. This morphological evidence supported the hypothesis that biosurfactant-mediated removal occurs through both chelation and co-precipitation mechanisms.

In the rhamnolipid soil washing study (*Pseudomonas* sp. biosurfactant for Cd, Cu, and Pb removal), scanning electron microscopy with EDS provided visual and elemental evidence of heavy metal extraction. SEM micrographs of the soil before and after treatment showed that originally “granular” metal-bearing deposits had disappeared; the biosurfactant-treated soil particles appeared smoother and pitted with newly formed pores. Energy-dispersive X-ray spectroscopy confirmed a significant drop in surface concentrations of Cd, Cu, and Pb after biosurfactant treatment. These SEM/EDS results indicated that the biosurfactant effectively dissolved or desorbed metal compounds from the soil matrix, correlating with the improved cleanup observed [98].

Together, these recent case studies underscore the central role of spectroscopic and microscopic techniques in characterizing biosurfactant–metal interactions. They not only confirm the involvement of specific functional groups and binding mechanisms but also reveal morphological and structural changes that are critical for understanding and optimizing biosurfactant-mediated recovery processes. These analytical insights are instrumental in advancing biosurfactant-based technologies toward more efficient and sustainable applications in critical mineral recovery.

## 8. Application of the Green Chemistry Metrics Framework to Biosurfactant-Based Recovery Processes

### 8.1. Application of Green Chemistry Framework to Recovery from Mining Waste

Applying Green Chemistry Metrics to mining waste processing highlights strong environmental advantages for biosurfactant-based approaches. Mine tailings and waste rocks exist in large volumes with relatively low metal concentrations, making conventional extraction both resource-intensive and hazardous. By contrast, biosurfactant leaching minimizes the use of aggressive chemicals, leading to a markedly smaller environmental footprint [99]. For example, rhamnolipid biosurfactants have been shown to effectively extract heavy metals (Ni, Cu, and Cd) from mining waste (even deep-sea tailings) under various conditions, while being inherently biodegradable and low in toxicity [100]. This aligns with Green Chemistry principles by reducing persistent pollution and allowing any residual reagents to degrade naturally instead of generating toxic sludge.

Quantitative green metrics underscore these benefits. Replacing strong mineral acids with biosurfactants can dramatically cut waste generation. Case analyses suggest biosurfactant-based processes achieve an Environmental Factor (E-factor) on the order of only 30–50 (kg of waste per kg of metal recovered), compared to E-factors of 150–300 for conventional acid leaching [99]. This corresponds to roughly an 80% reduction in waste, largely because biosurfactants avoid the extensive neutralization and precipitation steps that produce bulk chemical waste in acid treatments. The water footprint is likewise improved: biosurfactant leaching typically requires milder conditions and produces less contaminated water. In practice, this has translated to using about half the water per unit metal recovered, since fewer rinse and treatment cycles are needed [99]. Energy requirements are also lower, as biosurfactant-based extraction can often be performed at ambient temperature and pressure, unlike some hydrometallurgical processes that demand heating or pressurization. Notably, in conventional rare-earth extraction, chemical reagents (especially acids) contribute nearly 40% of the total greenhouse gas emissions and significant energy usage [67]. Biosurfactant methods eliminate most of these mineral acids, directly reducing the carbon footprint and energy consumption of the process.

In terms of technical feasibility, biosurfactant-based recovery from mining wastes is promising and has already seen successful case studies at the laboratory and pilot scale. One study achieved 84–90% recovery of copper from low-grade tailings by bioleaching with a microbial consortium enhanced by elemental sulfur (to generate biosurfactant-like organic acids in situ). The leachate was then treated biologically to selectively precipitate the copper, avoiding conventional lime neutralization sludge. The researchers noted that bio-processing these tailings would confer both environmental and economic benefits [101]. Importantly, biosurfactant leaching can often be conducted in existing waste impoundments or through in situ heap leaching, reducing the need for new infrastructure. This in situ applicability is a significant advantage at legacy mine sites—metals can be recovered while simultaneously remediating contamination, with minimal disturbance [100]. Biosurfactant-based processes operate at near-neutral pH in many cases, so equipment corrosion and material handling challenges are reduced compared to strong-acid processes. There are still practical challenges (e.g., longer leaching times to reach high recovery, and potential biosurfactant losses due to adsorption of minerals), but ongoing research is addressing these issues by optimizing biosurfactant formulas and delivery methods. Overall, Green Chemistry metrics favor biosurfactant-driven mining waste valorization, with dramatically lower hazardous waste output and energy use, and studies have demonstrated that these greener processes can extract valuable metals efficiently. The convergence of environmental benefits and technical viability make biosurfactant leaching an attractive alternative for sustainable mining waste management.

### 8.2. Application of Green Chemistry Framework to Spent Battery Recovery

Applying the Green Chemistry Metrics Framework to spent lithium-ion battery recycling reveals a more nuanced picture. Biosurfactant-based methods offer significant environmental advantages in principle, but they currently face limitations in processing efficiency and rate. On the positive side, using biosurfactants or other bio-based leaching agents in battery recycling can eliminate the need for concentrated acids and organic solvents, drastically reducing hazardous emissions and residues [67]. Greenhouse gas emissions are also lowered.

Pyrometallurgical battery recycling, which melts the battery materials in a furnace, is extremely energy-intensive and has a large carbon footprint; hydrometallurgical (acid-based) routes are somewhat less energy-intensive but still carry a significant burden [102]. In contrast, a bio-based leaching process operates at mild conditions can be significantly cleaner. A recent assessment showed that innovative recycling approaches (e.g., electrochemical or bio-based systems) could reduce overall energy use and GHG emissions by ~30% compared to the status quo [87]. The E-factor improvement is likewise notable: while traditional hydrometallurgy might generate on the order of 80–150 kg of waste per kg of metals recovered (due to acid fumes scrubbed solutions and neutralization sludges), a biosurfactant-enhanced process has been projected to bring that down to around 20–40 kg waste per kg product [87]. This 70–75% reduction in waste arises from avoiding lime neutralization steps and enabling reagent recycling. Furthermore, because biosurfactants do not produce noxious fumes, there is no need for off-gas scrubbing systems (which create secondary waste). For instance, the use of benign leaching agents prevents the release of hazardous gases like SO_2_, Cl_2_, and NO_X_ that can occur in conventional battery recycling when treating fluorinated binders or electrolytes [87].

In summary, biosurfactant-based recycling of spent batteries offers clear environmental benefits—greatly reduced hazardous waste generation, lower toxicity, and milder operating conditions—when benchmarked with Green Chemistry metrics. This approach could play a key role in making battery recycling more sustainable by shrinking the process’s carbon and water footprints and avoiding toxic outputs. However, technical feasibility remains an active area of research.

## 9. Future Directions and Challenges

### Research Gaps and Opportunities

This review has identified several critical research gaps that must be addressed to advance biosurfactant-based recovery of critical minerals. Standardized testing protocols for evaluating biosurfactant performance in metal recovery applications are lacking, limiting direct comparison between studies. The development and adoption of consistent methodologies aligned with the Green Chemistry Metrics Framework would facilitate more rigorous assessment and optimization [103].

Comprehensive life cycle assessments of biosurfactant-based recovery processes remain scarce, with most studies focusing on laboratory-scale evaluations of specific process steps rather than complete process chains. Expanding assessment boundaries to include biosurfactant production, waste preprocessing, and downstream metal purification would provide more realistic evaluation of overall sustainability impacts [104].

Biosurfactant recycling strategies require further development to improve both economic viability and environmental performance. Current approaches for recovering and reusing biosurfactants after metal extraction typically achieve recycling rates of 60–80%, with significant variation between applications [105]. Innovations in membrane separation, selective precipitation, and immobilization technologies offer promising pathways for enhancing recycling efficiency.

While current biosurfactants show preferential binding to certain metals, developing strategies to enhance selectivity for specific critical minerals, particularly lithium, cobalt, and rare earth elements, remains a significant challenge. Approaches including mixed biosurfactant systems, functionalized biosurfactants, and optimized process conditions show promise for addressing this challenge [106].

## 10. Conclusions

This review has introduced a comprehensive Green Chemistry Metrics Framework for evaluating biosurfactant-based recovery of critical minerals from waste materials. The framework provides a systematic approach to assessing environmental impact, resource efficiency, analytical performance, technical feasibility, and economic viability of these processes. By integrating multiple assessment domains, the framework enables holistic evaluation of sustainability performance and identification of optimization opportunities.

The application of the framework to existing studies reveals that biosurfactant-based recovery approaches offer significant potential advantages over conventional methods, particularly in terms of reduced environmental impact, lower toxicity, and enhanced sustainability [107]. E-Factors are typically 70–85% lower than conventional methods, carbon footprints are reduced by 50–75%, and dramatic reductions in toxicity metrics demonstrate the strong environmental case for biosurfactant-based approaches.

The economic viability of biosurfactant-based recovery is improving as production technologies advance and environmental externalities are increasingly recognized [108]. Applications involving high-value metals and/or highly hazardous conventional reagents already show compelling economic cases for biosurfactant-based approaches. As production costs continue to decrease and regulatory frameworks evolve to better account for environmental impacts, the economic case is expected to strengthen across a broader range of applications.

The Green Chemistry Metrics Framework introduced in this review provides a valuable tool for the objective evaluation of different biosurfactant-based recovery processes, meaningful comparison with conventional recovery methods, systematic identification of optimization opportunities, guiding research and development toward greener and more sustainable approaches, and facilitating regulatory acceptance and industrial adoption.

Advancing biosurfactant-based technologies for critical mineral recovery represents a promising pathway toward more sustainable resource management and circularity in the critical mineral supply chain. By applying green chemistry principles through the proposed metrics framework, researchers and industry practitioners can systematically improve these technologies to address the twin challenges of resource scarcity and environmental protection.

Future research should focus on addressing the identified gaps and challenges, particularly in areas of selectivity enhancement, biosurfactant recycling, and scale-up demonstration. The integration of biosurfactant-based recovery with complementary technologies including synthetic biology, nanotechnology, and artificial intelligence offers particularly promising directions for advancement. The continued evolution of these technologies, guided by the Green Chemistry Metrics Framework, has the potential to transform waste materials from environmental liabilities into valuable resources for the clean energy transition.

## Figures and Tables

**Table 1:** Key properties of biosurfactants relevant to critical mineral recovery [46,47,48,49,50,51,52,53,54,55,56,57,58,59,60,61,62,63,64,65].

Property	Rhamnolipids	Surfactin	Sophorolipids	Significance for Metal Recovery
Structural Properties				
Molecular Weight (Da)	500–900	1000–1100	600–800	Affects diffusion and interaction with metals
Critical Micelle Concentration (mg/L)	5–200	10–150	40–150	Lower CMC indicates greater efficiency at lower concentrations
Surface Tension Reduction (mN/m)	25–30	27–32	30–35	Lower values indicate better interfacial activity
Environmental Properties				
Optimal pH Range	4.0–8.0	5.0–10.0	4.0–7.5	Affects metal binding capacity and stability
Temperature Stability (°C)	−20 to 120	0 to 100	−10 to 130	Determines applicability under various process conditions
Biodegradability (%)	>90 (28 days)	>80 (28 days)	>95 (28 days)	Higher values indicate lower environmental persistence
Aquatic Toxicity (EC50, mg/L)	50–150	40–120	100–300	Higher values indicate lower toxicity
Production Properties				
Primary Microbial Sources	*Pseudomonas aeruginosa*	*Bacillus subtilis*	*Starmerella bombicola*	Affects production methods and sustainability
Production Yield (g/L)	10–100	0.1–5	100–400	Higher yields reduce production costs
Production Time (days)	4–7	2–5	5–10	Shorter times improve production efficiency
Potential for Waste Substrate Use	High	Medium	Very High	Affects overall sustainability of production

**Table 2:** Specific recovery efficiencies for critical minerals from different waste streams.

Metal	Waste Type	Method Type	Leaching Method	Conditions	L/S Ratio (*v*/*wt*)	Initial Metal Concentration	Recovery (%)	Reference
Copper (Cu)	Contaminated sediment	Biosurfactant	Rhamnolipid washing (0.5%)	pH~7; Ambient temp; 1 h	NR	NR	65%	[81,86]
Copper (Cu)	Printed Circuit Boards (PCBs)	Biosurfactant	Biosurfactant-enhanced bioleaching	Neutral pH; 30 °C; 14 days	NR	~20–30 wt% Cu	<53%	[82,86]
Copper (Cu)	Printed Circuit Boards (PCBs)	Conventional	Cl_2_ gas in 4M HCl solution	pH < 1; 45 °C; 100 min	NR	~20–30 wt% Cu	87.2%	[83]
Zinc (Zn)	Contaminated sediment	Biosurfactant	Sophorolipid washing (4%)	pH~7; Ambient temp; 1 h	NR	NR	60%	[81]
Zinc (Zn)	Printed Circuit Boards (PCBs)	Biosurfactant	Biosurfactant-enhanced bioleaching	Neutral pH; 30 °C; 14 days	NR	NR	~48%	[82]
Zinc (Zn)	Printed Circuit Boards (PCBs)	Conventional	Cl_2_ gas in 4M HCl solution	pH < 1; 45 °C; 100 min	NR	NR	>90%	[83]
Nickel (Ni)	Spent refinery catalyst	Biosurfactant	Rhamnolipid leaching (0.4 mg/L)	pH~7; 30 °C; 7 days	NR	2–10 wt% Ni	30%	[85]
Nickel (Ni)	Printed Circuit Boards (PCBs)	Biosurfactant	Biosurfactant-enhanced bioleaching	Neutral pH; 30 °C; 14 days	NR	5–10 wt% Ni	<48.5%	[82]
Nickel (Ni)	Lithium-ion battery waste (“black mass”)	Conventional	H_2_SO_4_ + H_2_O_2_ leaching	2M H_2_SO_4_ + 5% H_2_O_2_; 80 °C; 60 min	10:1	~5–30 wt% Ni	~100%	[84]
Lead (Pb)	Printed Circuit Boards (PCBs)	Biosurfactant	Biosurfactant-enhanced bioleaching	Neutral pH; 30 °C; 14 days	NR	1–5 wt% Pb	~0.5%	[82]
Lead (Pb)	Printed Circuit Boards (PCBs)	Conventional	Cl_2_ gas in 4M HCl solution	pH < 1; 45 °C; 100 min	NR	1–5 wt% Pb	>90%	[83]
Lithium (Li)	Lithium-ion battery waste (“black mass”)	Biosurfactant	Rhamnolipid leaching	pH 6.0–7.0; Room temp; ~48 h	NR	5–7 wt% Li	~75%	[87]
Lithium (Li)	Lithium-ion battery waste (“black mass”)	Conventional	H_2_SO_4_ + H_2_O_2_ leaching	2M H_2_SO_4_ + 5% H_2_O_2_; 80 °C; 60 min	10:1	5–7 wt% Li	~100%	[84]
Cobalt (Co)	Lithium-ion battery waste (“black mass”)	Biosurfactant	Rhamnolipid leaching	pH 4.0–5.0; Room temp; ~48 h	NR	~5–20 wt% Co	~90%	[87]
Cobalt (Co)	Lithium-ion battery waste (“black mass”)	Conventional	H_2_SO_4_ + H_2_O_2_ leaching	2M H_2_SO_4_ + 5% H_2_O_2_; 80 °C; 60 min	10:1	~5–20 wt% Co	~100%	[84]
Gold (Au)	Printed Circuit Boards (PCBs)	Biosurfactant	Sophorolipid-enhanced leaching	pH 3.0–4.0; Room temp; Time NR	NR	~0.01–0.03 wt% Au	65–80%	[83]

Note: NR = Not Reported. Recovery efficiency ranges are compiled from published studies and represent optimal conditions achieved in laboratory settings. Industrial-scale applications may show different efficiency ranges [81,82,83,84,85,86,87].

**Table 3:** Comparative assessment of metal recovery methods based on Green Analytical Chemistry (GAC) principles.

GAC Principle	Hydrometallurgical	Pyrometallurgical	Biosurfactant-Based Recovery	Alignment with Green Analytical Chemistry (GAC)	
Solvent Use (g/sample or L/kg)	2–5 L strong acids per kg waste [45]		None (uses solid flux/reductants) [45]	0.5–5 g/L biosurfactant in aqueous media [94]	Biosurfactant-Based (lowest toxicity, low dosage)
Energy Consumption (kWh/kg)	8–9 kWh/kg [45]		~4.7–10 kWh/kg [45]	0.1–1.0 kWh/kg (mostly mixing) [94]	Biosurfactant-Based (lowest energy)
Reagent Toxicity	High (corrosive acids, volatile solvents) [45]		High (metal vapors, dioxins, and toxic gases) [45]	Low (biodegradable, non-volatile) [95]	Biosurfactant-Based
Waste Generation (E-Factor)	80–300 kg of waste per kg of recovered metal (includes acid leachates and sludge) [96]	3–5 kg of slag and dust per kg of recovered metal [45]	20–45 kg of waste per kg of recovered metal [95]	Biosurfactant-Based (least waste, biodegradable)	
Biodegradability of Inputs	Non-biodegradable: mineral acids, solvents [45]	Not applicable: inorganic slag [45]	Highly biodegradable: >80–95% within 28 days [95]	Biosurfactant-Based	
Sample Preparation Intensity	High: pH control, solid–liquid separation, neutralization, and washing [91]	Moderate: thermal pretreatment, slag separation, and gas handling [92]	Low: aqueous mixing; minimal pH adjustment; and no toxic cleanup steps [97]	Biosurfactant-Based	
Operator Safety Hazards	High: corrosive acids, toxic fumes [92]	High: >1000 °C, metal vapors, and toxic gases; gas scrubbing needed [92]	Low: ambient conditions, non-toxic reagents [97]	Biosurfactant-Based (safest)	

Note: Implementation strategies represent recommended approaches for enhancing the green chemistry profile of biosurfactant-based recovery methods [45,94,95,96,97].

## Data Availability

No new data were created.
